# A Prospective Analysis of Quality of Life and Toxicity Outcomes in Treating Early Breast Cancer With Breast Conservation Therapy and Intraoperative Radiation Therapy

**DOI:** 10.3389/fonc.2018.00545

**Published:** 2018-12-03

**Authors:** Michael Sosin, Surupa Sen Gupta, Jessica S. Wang, Corinne D. Costellic, Aiste Gulla, Alex J. Bartholomew, Suzanne C. O'Neill, Elizabeth M. Hechenbleikner, Brian T. Collins, Sonali Rudra, Sean P. Collins, Krysta M. Chaldekas, Sulakshana Seevaratnam, Russell C. Langan, Shawna C. Willey, Eleni A. Tousimis

**Affiliations:** ^1^Department of Surgery, Division of Breast Surgery, Medstar Georgetown University Hospital, Washington, DC, United States; ^2^Department of Plastic Surgery, Medstar Georgetown University Hospital, Washington, DC, United States; ^3^Georgetown Lombardi Comprehensive Cancer Center, Washington, DC, United States; ^4^Department of Radiation Medicine, Medstar Georgetown University Hospital, Washington, DC, United States

**Keywords:** quality of life, radiation, intraoperative radiation, toxicity, breast conservation

## Abstract

**Introduction:** Intraoperative radiation therapy (IORT) is a minimally invasive radiation option for select patients with early stage breast cancer. This prospective, single institution, pilot study summarizes patient-reported quality of life (QoL) outcomes and clinician-reported toxicity following IORT following breast conservation therapy.

**Methods:** Forty-nine patients were enrolled in a prospective study from 2013 until 2015 to assess QoL and toxicity following breast conservation therapy and IORT. Nine patients did not meet criteria for IORT alone on final pathology and required whole breast irradiation afterwards. These patients were evaluated separately. Validated QoL questionnaires were provided to patients at 1-week, 1-month, and subsequent 6-month intervals for 2 years. Radiation-related toxicity symptoms were evaluated by clinicians at the same time intervals. Likert scale responses were converted to continuous variables to depict patient-reported and clinician-reported outcomes.

**Results:** Outcomes were analyzed as weighted averages of the Likert scale for each symptom. Responses for negative QoL symptoms ranged largely from 0 (none) to 2 (moderate). Responses for positive QoL symptoms ranged largely from 3 (quite a bit) to 4 (very much). Seventy-five percent of patients developed a toxicity; however, 99% of the toxicities were grades 1 and 2. All toxicities demonstrated a downward trend over time, with the exception of breast fibrosis and nodularity, which increased over time. There were no local recurrences upon 2-year follow up.

**Conclusion:** Early stage breast cancer treated with IORT yields favorable QoL outcomes and minimal toxicity profiles with adequate short-term local control.

## Highlights

- Patients undergoing IORT following BCT consistently report strong emotional well-being and social support.
- Patients undergoing IORT following BCT shows mild to moderate limitations on activities of daily living.
- The severity and incidence of IORT-induced toxicities are minimal and infrequent, respectively.

## Introduction

Conventional radiation therapy after breast conservation therapy (BCT) for early stage breast cancer involves 3–5 weeks of whole breast irradiation (WBI) followed by a 1-week boost dose to the lumpectomy cavity is select cases (1). Although BCS with adjuvant WBI has equivalent overall survival compared to mastectomy, patients may experience challenges adhering to the prolonged duration of therapy. The logistical difficulties with this regimen may ultimately compromise a patient's quality of life (QoL), especially for those living in remote areas that may lack easy access to radiation therapy. The stress and inconvenience of radiotherapy may lead to non-compliance and insufficient treatment (2). The need for WBI has been shown to impact patients' decision to undergo a mastectomy instead (3). The advent of intraoperative radiation therapy (IORT) has shown promise in mitigating the pitfalls of WBI.

Targeted intraoperative radiation therapy via the INTRABEAM™ radiotherapy system is a form of accelerated partial breast irradiation that delivers ~20 Gy of targeted radiation in a single dose to the lumpectomy cavity at the time of surgery (1, 4, 5). This allows for the local therapy to be completed at one time, eliminating the need for several weeks of daily treatment. The rationale behind IORT is to target the tumor bed, the site at highest risk of recurrence, with proper dose homogeneity while sparing normal tissue (1–3, 6). The international randomized phase III TARGIT-A trial confirmed the oncologic safety of IORT by demonstrating local recurrence rates to be non-inferior to those of standard WBI at 5 years (2, 5, 7). IORT's unique ability to spare the healthy tissue from radiation further decreases the risk of developing high-grade radiation-related toxicity (8–10).

The potential benefits of IORT are numerous, but the impact on patient-reported QoL and the toxicity profile remain variable (6, 11–13). While IORT provides a shorter duration of therapy, if patients feel that this modality is uncomfortable, painful, or anxiety provoking, the advantages may be negligible. The aim of this study is to assess patient-reported QoL and clinician-reported toxicity outcomes for patients with early stage breast cancer undergoing IORT at a single institution. We hypothesize that patients receiving IORT at our institution would have favorable QoL responses and minimal toxicity profiles.

## Methods

Following approval from the Medstar Georgetown University Institutional Review Board, IORT was initiated at MedStar Georgetown University Hospital in January 2013. The initial inclusion criteria (Table 1) were based on both the TARGIT-A trial and the ASTRO consensus guidelines for partial breast irradiation (5, 14). In 2015, the inclusion criteria were adjusted to include tumors up to 3 cm in size and surgical margins of 1 mm, as these criteria fall outside of the ASTRO PBI consensus guidelines. However, margins up to 1 mm and small T2 tumors were captured in the TARGIT-A trial for IORT as the single treatment modality. The INTRABEAM™ radiotherapy system (Carl Zeiss, Oberkochen, Germany) was used to perform IORT. All patients received 50 kV of low energy photons in a single 20 Gy fraction to the lumpectomy cavity at the time of surgery.

**Table 1:** Inclusion criteria for IORT.

**Eligibility criteria**
Age ≥ 50
Tumor size ≤ 2 cm^*^
Histology of pure infiltrating ductal carcinoma
No lymphovascular invasion
Low/intermediate grade
ER+/PR+ receptor status
No clinical or histologic nodal disease (N0)
Unifocal disease
Surgical margins ≥ 2 mm^†^

* *Amended in 2015 to tumors ≤ 3 cm*.

† *Amended in 2015 to surgical margins ≥ 1 mm*.

All eligible patients were identified by the treating surgeon and/or radiation oncologist at the time of consultation. A dedicated research assistant approached all interested patients and obtained informed consent prior to participation in the study. Patients were given multiple validated QoL questionnaires during their 1-week, 1-month, 6-month, 1-year, 1.5-year, and 2-year follow-up appointments. Questionnaires included the Euro-QoL (EQ-5D), Functional Assessment of Cancer Therapy-Breast (FACT-B Version 4), Functional Assessment of Chronic Illness Therapy (FACIT) Fatigue Scale (Version 4), and European Organization for Research and Treatment of Cancer (EORTC) Quality of Life Questionnaire Breast Cancer Module (QLQ-BR23).

The EQ-5D questionnaire is a descriptive profile with two components. The first provides patient-rated health status across the domains of mobility, self-care, usual activities, and anxiety/depression. The second is a visual analog scale (VAS) providing a single index value for patient perceived current health status, with 0 being “the worst health you can imagine” and 100 being “the best health you can imagine.” The FACT-B survey is a 42-item compilation of general questions divided into the domains of physical, social, emotional, and functional well-being. The FACIT Fatigue Scale involves 13 questions related to fatigue level. The QLQ-BR23 module consists of symptom scales for pain in the ipsilateral extremity, edema in the ipsilateral extremity, pain in the affected breast, edema in the affected breast, oversensitivity in the affected breast, skin problems in the affected breast, and difficulty with arm movement. For all of the questionnaires, a Likert scale of 0 to 4 was used to correlate with the following: 0 = not at all, 1 = mild, 2 = moderate, 3 = quite a bit, 4 = very much. Responses of 0 to 2 for negative questions (“I have a lack of energy”) and responses of 3 to 4 for positive questions (“I feel close to my friends”) were considered favorable outcomes.

Radiation-related toxicity was evaluated by physicians at the same time intervals. Each evaluation was performed and recorded by the operating surgeon. The evaluations were performed using the Common Terminology Criteria for Adverse Events (CTCAE) Version 4.03 form. Patients were assessed for dermatitis, breast pain, breast swelling, fibrosis, palpable nodularity, telangiectasias, breast indentation, ulceration, hematoma, seroma, and wound infection. Symptoms were graded on a Likert scale of 0 to 5 as follows: 0 = no symptoms, 1 = mild symptoms, 2 = moderate symptoms not affecting activities of daily living (ADL), 3 = severe symptoms affecting ADL, 4 = disabling symptoms interfering with ADL, 5 = death. In order to quantitatively evaluate changes over time, the qualitative data was converted to continuous variables by conversion to weighted means for each assessment tool.

## Results

Fifty-three patients were eligible to receive IORT, with 49 patients electing to enroll in the study. Mean duration of IORT treatment was 22.3 min with a range of 17.5 to 45.3 min. Nine patients did not meet criteria on final pathology for IORT alone and proceeded to receive WBI after IORT. These patients were removed from the study population and examined separately. Reasons for WBI included positive margins (*n* = 4), disease multifocality (*n* = 3), and high-grade pathology/lymphovascular invasion (*n* = 2). Patient demographics, cancer stage, final pathology (based on the American Joint Committee on Cancer 7th ed.), and adjuvant treatments for the IORT group and the IORT plus WBI group are listed in Table 2. All patients underwent sentinel lymph node biopsy, and no patients had an axillary lymph node dissection. There were no local recurrences in any of the 49 participants.

**Table 2:** Demographics, cancer stage, pathology, and adjuvant treatments for patients receiving IORT vs. patients receiving IORT plus WBI.

**Demographics**	**IORT (N, %)**	**IORT plus WBI (N, %)**
n	40	9
Age (yrs)^*^	64.5 (50–83)	64.3 (50–77)
**RACE**		
White	30, 75.0%	6, 66.7%
Black	7, 17.5%	1, 11.1%
Other	3, 7.5%	2, 22.2%
Married	18, 45.0%	5, 55.6%
Higher Education	19, 47.5%	5, 55.6%
Employed	18, 45.0%	5, 55.6%
**CANCER STAGE**		
IA	33, 82.5%	7, 77.7%
IIA	2, 5.0%	2, 22.2%
Unknown	5, 12.5%	0, 0.0%
**FINAL PATHOLOGY**		
IDC	8, 20.0%	1, 11.1%
IDC with DCIS	25, 62.5%	8, 88.9%
Other	7, 17.5%	0, 0.0%
Endocrine Therapy^†^	33, 82.5%	8, 88.9%
Tamoxifen	9, 22.5%	3, 33.3%
Aromatase Inhibitor	22, 55.0%	5, 55.6%
Combination/Other	2, 5.0%	0, 0.0%
Chemotherapy	0, 0.0%	3, 33.3%
Axillary lymphadenectomy	

* *Data presented as mean (range)*.

† *Seven patients declined endocrine therapy*.

### Quality of life

**Figure 1:**
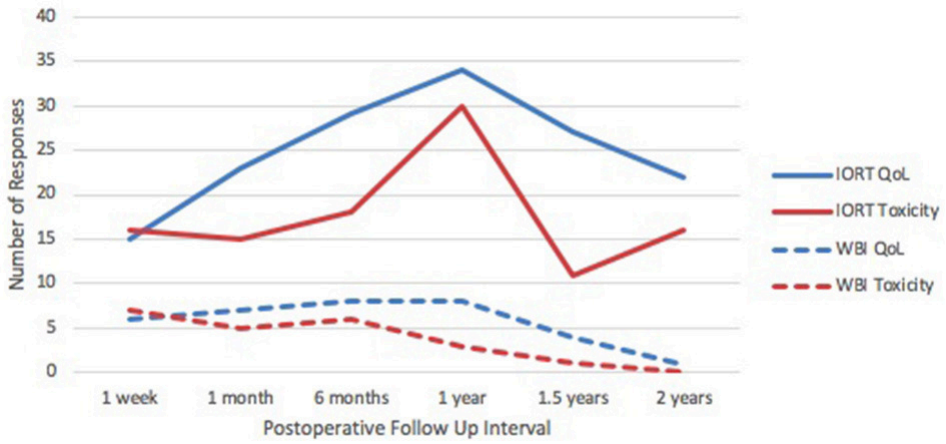
IORT Patient Response Rates. Response rates in QoL and toxicity for the IORT group were consistently higher than those in the IORT + WBI group. Peak response rate in the IORT group occurred at the 1-year postoperative mark.

**Figure 2:**
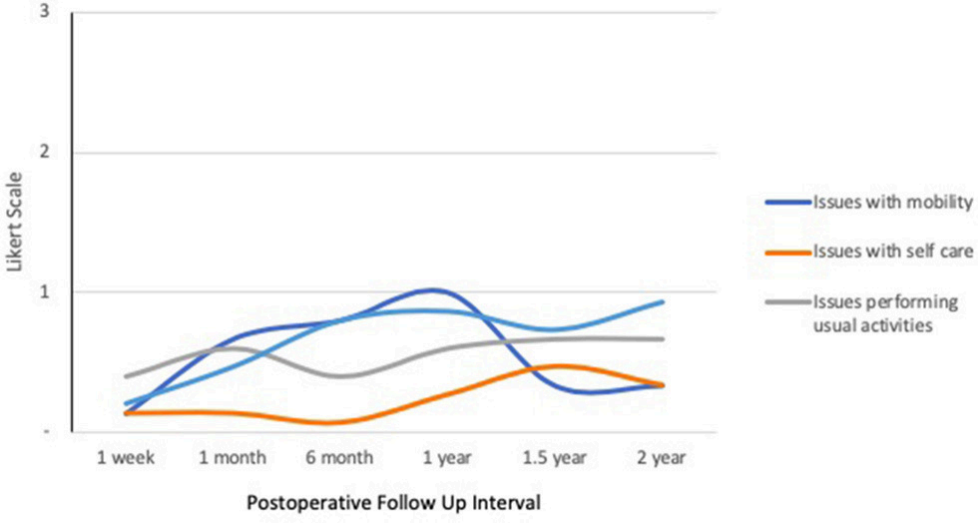
IORT Patient-Reported Health Status (EQ-5D). Patients reported scores between 0 and 1 across all symptoms, indicating “no issues” to “mild issues” on the Likert scale. Of note, anxiety/depression increased over time.

Quality of life response rates in the IORT group were lowest at 1-week follow up and highest at 1-year (Figure 1). The EQ-5D demonstrated that patients reported a mean self-health assessment score of 87.8 at 1 week and 77.0 at 6 months after surgery, respectively. The median self-health score across all time points was 81.7. In all categories across all time points, the responses were between 0 and 1, indicating “no issues” to “mild issues” on the Likert scale. Pertaining to mobility categories, the weighted average Likert score was lowest at 1 week (0.13) and highest at 1 year (1.00), as illustrated in Figure 2. Regarding self-care, the lowest average occurred at 6 months (0.07), and the highest at 1.5 years (0.47), with a slight uptrend in the second half of study. Regarding the performance of usual activities, the lowest average occurred at 1 week and 6 months (0.40), and the highest at 1.5 to 2 years (0.67). Anxiety and depression were lowest at 1 week (0.20) and highest at 2 years (0.93), demonstrating an upward trend over time.

**Figure 3:**
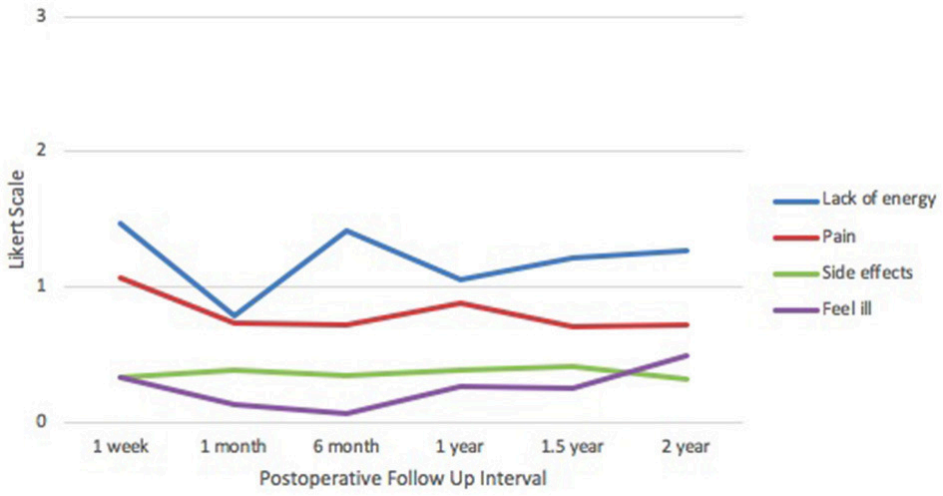
IORT Patient-Reported Physical Well-Being (FACT-B). Patients reported scores between 0 and 2 across all symptoms, indicating “no issues” to “moderate issues” on the Likert scale.

In the FACT-B+4 physical well-being domain (Figure 3), patients were most affected by lack of energy at 1 week postoperatively (1.47) and least affected at 1 month (0.78). Pain was highest at 1 week (1.07) and lowest at 1.5 years (0.70), hovering steadily between 0.70 and 0.90 from 1 month onward. Reported scores for side effects remained stable through all time points at ~0.35. Feelings of illness were lowest at 6 months (0.07) and highest at 2 years (0.50). Responses were between 0 and 2 across all categories and time points, correlating with feelings of “no issues” to “moderate issues,” respectively.

**Figure 4:**
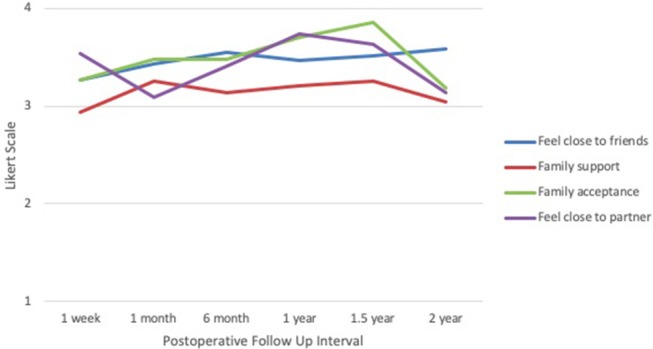
IORT Patient-Reported Social Well-Being (FACT-B). Patients reported scores largely between 3 and 4 across all symptoms, indicating “quite a bit” to “very much” on the Likert scale.

In the social well-being domain (Figure 4), mean scores for feeling close to friends were lowest at 1 week after surgery (3.27), with an upward trend reaching a peak at 2 years (3.59). Family support was lowest at 1 week (2.93) and highest at 1 month and 1.5 years (3.26). Family acceptance scores peaked at 1.5 years (3.85), followed by a trough at 2 years (3.18). Feelings of closeness to one's partner were lowest at 1 month (3.09) and highest at 1 year (3.74). Across all categories and time points, responses were mainly between 3 and 4, indicating “quite a bit” to “very much” on the Likert scale.

**Figure 5:**
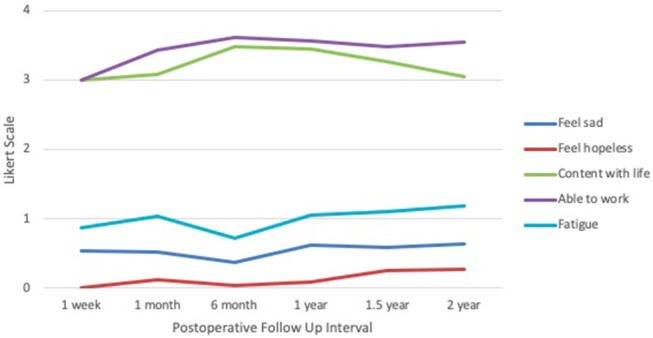
IORT Patient-Reported Emotional/Functional Well-Being (FACT-B) and Fatigue Level (FACIT). Patients reported scores largely between 0 and 1 across the negative symptoms, indicating “none” to “mild” on the Likert scale, and between 3 and 4 across the positive symptoms, indicating “quite a bit” and “very much”.

Within the emotional well-being section (Figure 5), average score for sadness were consistent at ~0.50 across all time points, with the lowest occurring at 6 months (0.38) and the highest at 2 years (0.64). Feelings of hopelessness remained low, with the nadir at 1 week (0.00) and the peak at 2 years (0.27). Feelings of contentment with life were lowest at 1 week (3.00) and highest at 6 months (3.48), declining again at 2 years (3.05). In the functional well-being domain, ability to work was lowest at 1 week (3.00) and highest at 6 months (3.62). Based on the FACIT survey, levels of fatigue were lowest at 6 months (0.72) and highest at 2 years (1.18). Categories of sadness, hopelessness, and fatigue received responses closest to 0 and 1, corresponding to “none” and “mild” on the Likert scale, respectively. Categories of contentment and ability to work received responses between 3 and 4, corresponding to “quite a bit” and “very much,” respectively.

**Figure 6:**
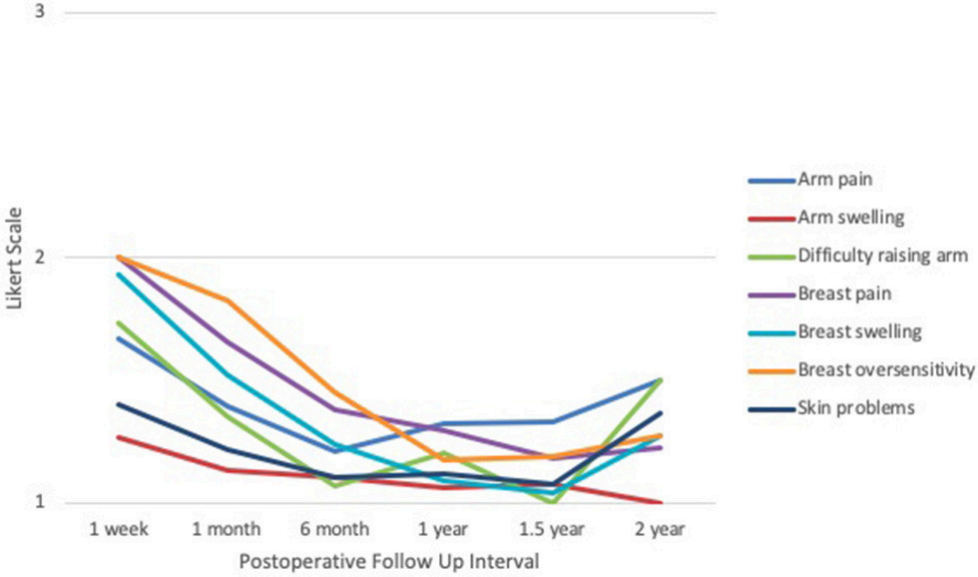
IORT Patient-Reported Symptoms (EORTC QLQ). Patients reported scores between 1 and 2 across all symptoms, indicating “mild” to “moderate” on the Likert scale. Of note, these symptoms appear to decline with time but experience a slight increase between 1.5 to 2 years.

Based on the EORTC QLQ patient-reported symptoms survey (Figure 6), arm pain scores were highest at 1 week (1.67) and lowest at 1 year (1.32). Arm swelling scores were highest at 1 week (1.27) and lowest at 2 years (1.00). Difficulty raising arm(s) was highest at 1 week (1.73) and lowest at 1.5 years (1.00). Breast pain was highest at 1 week (2.00) and lowest at 1.5 years (1.19). Similarly, breast swelling was highest at 1 week (1.93) and lowest at 1.5 years (1.04). Breast oversensitivity was highest at 1 week (2.00) and lowest at 1 year (1.18). Skin problems scored highest at 1 week (1.40) and lowest at 1.5 years (1.07). Nearly all categories followed a downtrend over time; however, scores for difficulty raising arm(s), skin problems, and breast swelling experienced a slight increase between 1.5 and 2 years. All responses across all categories and time points remained between 1 and 2, corresponding to “mild” and “moderate,” respectively.

### Toxicity

**Figure 7:**
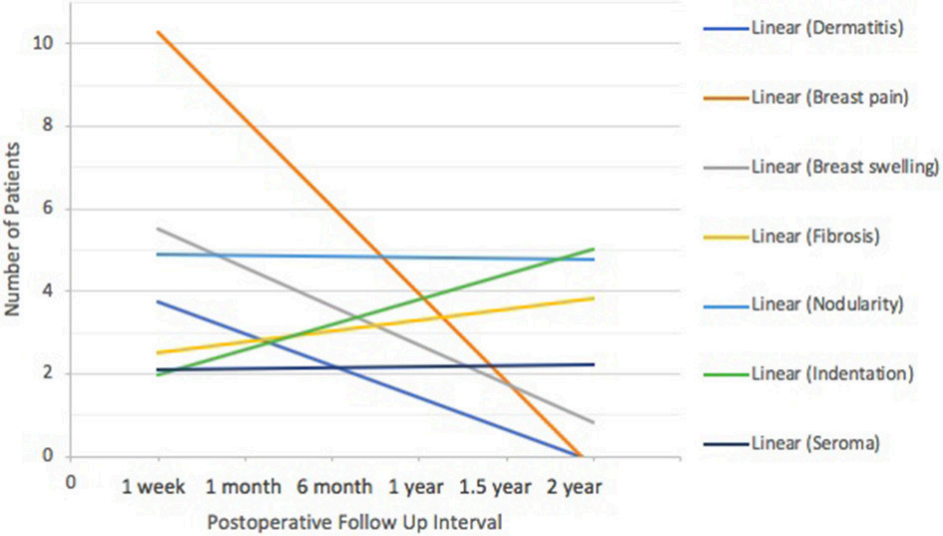
IORT Clinician-Reported Toxicity (CTCAE) Trends. Dermatitis, breast pain and breast swelling were most prevalent at 1 week to 1 month after surgery, followed by a downtrend over time. Seromas and breast nodularity remained steady. Breast fibrosis and indentation followed an uptrend as time progressed.

Post-treatment toxicities were evaluated and recorded by the surgeon of record at each time point. Response rates are depicted in Figure 1, and the total number of toxicities is listed in Table 3. Ninety-nine percent of reported toxicities were grades 1 and 2, and the remaining 1% were grade 3 (fibrosis and indentation). There were no grade 4 or 5 toxicities. Dermatitis (*n* = 6) and breast pain (*n* = 10) were most prevalent at 1 week to 1 month after surgery, followed by a downtrend over time (Figure 7). Breast swelling followed a similar trend, with the peak incidence at 1 week (*n* = 6) postoperatively. In contrast, fibrosis had the lowest frequency at 1 week (*n* = 1), followed by higher frequencies as time progressed (*n* = 5). Breast nodularity and indentation were both highest at 1 year (*n* = 9, *n* = 8 respectively), with indentation following an upward trend as time progressed. Seroma formation was highest at 1 month (*n* = 4) without any obvious trend. Not depicted in Figure 7 were three hematomas that resolved without intervention and a wound infection at the 1-week mark that resolved with antibiotic therapy. There were no skin ulcerations or telangiectasias throughout the study period.

**Table 3:** Clinician-reported toxicity: Total toxicities recorded in the IORT group (*n* = 40).

	**1 week**	**1 month**	**6 months**	**1 year**	**1.5 year**	**2 year**
Grade 1	29	41	14	31	10	18
Grade 2	5	5	0	3	1	0
Grade 3	0	0	0	0	0	2
Grade 4	0	0	0	0	0	0
Grade 5	0	0	0	0	0	0
Total	34	46	14	34	11	20

### IORT with additional whole breast irradiation

Response rates for QoL and toxicity surveys in the IORT plus WBI group (*n* = 9) are depicted in Figure 1. Given the small sample size, response rates dwindled to zero or one at the 2-year mark. The median overall health score was 72. Responses to the EQ-5D questionnaire were between 0 and 2 across all time points (Supplemental Figure 1), corresponding to “none” and “moderate,” respectively.

The same result was seen in the responses to the physical well-being domain of the FACT-B+4 survey (Supplemental Figure 2), with the exception of one outlier of a score of 3 in the pain category at the 2–year mark, corresponding to “quite a bit” on the Likert scale. The majority of responses in the social well-being domain stayed near 3 and 4, corresponding to “quite a bit” and “very much,” respectively. However, scores for closeness to partner fluctuated with drops at 1 month (2.14) and 2 years (1.00) (Supplemental Figure 3).

In the emotional/functional well-being domain, responses for sadness and hopelessness were consistently between 0 and 1 across all time points, corresponding to “none” and “mild,” respectively. However, fatigue scores showed an upward trend with the peak at 2 years (3.0). Most responses regarding contentment with life and ability to work hovered between 2 and 3, or “moderate” and “quite a bit,” respectively (Supplemental Figure 4).

All patient-reported symptoms from the EORTC QLQ survey scored approximately between 1 and 2, or “mild” and “moderate,” respectively. However, no obvious trends were seen (Supplemental Figure 5). All reported toxicities were grades 1 or 2, except for one grade 3 seroma at the 6-month mark (Table 4). All toxicity rates demonstrated a downward trend as time progressed with the exception of breast fibrosis and indentation.

**Table 4:** Clinician-reported toxicity: Total toxicities recorded in the IORT + WBI Group (*n* = 9).

	**1 week**	**1 month**	**6 months**	**1 year**	**1.5 year**	2 year^*^
Grade 1	13	11	3	8	3	–
Grade 2	1	0	1	3	0	–
Grade 3	0	0	1	0	0	–
Grade 4	0	0	0	0	0	–
Grade 5	0	0	0	0	0	–
Total	14	11	5	11	3	–

* *At the two-year mark, the number of respondents was zero*.

## Discussion

This study implemented IORT in patients with early breast cancer undergoing BCT and prospectively assessed patient-reported QoL, evaluated toxicity of therapy, and tracked short-term local recurrence rates. The findings support a favorable QoL profile across multiple domains and assessment tools for patients undergoing BCT with IORT. Additionally, our early experience with IORT revealed low toxicity rates with encouraging oncologic outcomes.

Consistent with historical trends in breast cancer therapy, the impetus for minimally invasive therapy has brought BCT to the forefront of early breast cancer treatment. The introduction of BCT followed by WBI was received with skepticism until multiple randomized clinical trials, including the National Surgical Adjuvant Breast and Bowel Project (NSABP), demonstrated comparable survival and local recurrence rates to mastectomy therapy (15–17). Several studies have shown encouraging rates of quality of life associated with WBI, but the disruption a patient's quality of life and breast changes secondary to radiation confer a need for improvement (18–22). Whelan et al. conducted a randomized control study delineating a statistically significant impairment of QoL in patients receiving WBI, but radiation techniques were less advanced than current radiation delivery (21) Lee et al. attributed impaired quality of life due to transient skin and subcutaneous skin toxicity, which resolved to baseline scores by 7 months, and although the results are favorable, the patients did not undergo further follow up beyond 7 months (18). The impact of WBI on QoL remains variable amongst different experiences. Wallace et al. have shown that high dosage and long schedules for WBI is associated with a greater disruption of private life and a less positive outlook on the completion of radiotherapy treatment compared to lower radiation dosage and shorter treatment schedules (20). On the other hand, Xaio et al. found that patients undergoing WBI did not demonstrate an association with skin toxicity and QoL scores. However, elevated BMI and high perceived stress levels correlated with lower SF-36 scores, and they noted that low scores persisted to the 1-year mark (19). Long-term follow up of 15 years shows a progressive increase in QoL scores (22). Amidst much of the data surrounding WBI, there remains a component of distress, inconvenience, and toxicity that invariably affects patients undergoing breast conservation therapy.

The shift toward BCT from mastectomy has generated interest in delivering a more targeted form of adjuvant radiation therapy from traditional WBI. In 2010, the TARGIT-A trial demonstrated that IORT was non-inferior to WBI in patients with early stage breast cancer undergoing BCT. The difference in local recurrence rates (IORT = 1.2% vs. WBI = 0.95%) at the 4-year mark was not statistically significant (5, 23). Follow-up reporting in 2014 demonstrated a 2% increased risk of local recurrence with IORT compared to WBI, but a 2 to 3% decrease in overall mortality at the 5-year mark (2). Although oncologically non-inferior, patient-reported QoL outcomes are paramount for such novel therapy to gain popularity within both the public and medical communities. Initial radiation-related QoL parameters after IORT and WBI were reported from a single center participating in the TARGIT-A trial (13). Results indicated that IORT patients reported less pain, fewer breast/arm symptoms, and fewer restrictions in daily activities compared to WBI patients (13). Similarly, our study demonstrated low rates of negative side effects throughout the 2-year period. Breast and arm symptoms were highest at 1-week after surgery and followed a downward trend as time progressed, as expected during the postoperative healing course. However, there was a slight increase in these side effects at the 1.5 to 2-year marks, which may be attributed to the low response rate or related to patient expectations of being symptom-free by this point in recovery course. Rates relevant to issues performing usual activities, sadness, hopelessness, and fatigue remained consistently low throughout the follow-up period; these results were supplemented with high rates of contentment with life and ability to work. Patients demonstrated excellent support systems as demonstrated by high rates of closeness to friends and family throughout the study. Interestingly, our findings identified a vulnerable period for patient function at the 1.5 to 2-year time points. Feelings of illness, anxiety/depression, family acceptance, and closeness to partner during this time frame deviated from the expected trend, which coincided with the aforementioned deviations in breast and arm symptoms. This finding may be due to the side effects of endocrine therapy (selective estrogen receptor modulators or aromatase inhibitors), which was administered in 82.5%. Furthermore, the 1 to 1.5-year time point may coincide with less frequent office visits and perception of completion of treatment, yet persistent negative side effects and the psychological impact may be magnified during this period.

In addition to achieving local control with IORT, minimizing toxicities is critical to the adoption of this new treatment protocol by oncologists (surgical, medical, and radiation) and patients. Side effects can have a devastating impact on patient QoL, to the point of tipping the risk-benefit scale toward the unfavorable side (8). The IORT INTRABEAM™ dose of 20 Gy to the tumor bed is equivalent to 5–6 weeks of WBI totaling 50 to 60 Gy (23). The primary concern with IORT is the increased risk of late toxicity with a large one-time dose. However, as IORT is delivered directly into the lumpectomy cavity, the negative effects of radiation to the skin and underlying breast tissue appear to be diminished (8, 23). Studies have shown that erythema, telangiectasia, and hyperpigmentation appear less frequently and are less severe following IORT when compared to WBI (12, 23). Our results support this conclusion as rates of wound infection (*n* = 1), telangiectasia (*n* = 0) and ulceration (*n* = 0) remained negligible in the IORT patients throughout the study period. In addition, our rates of dermatitis were highest within the first postoperative month but decreased to a negligible amount by the sixth month. More commonly reported IORT-related toxicities are fat necrosis, seroma, and breast fibrosis or retraction (23). Twenty-two percent (*n* = 9) of IORT patients developed seromas; all incidences were grade 2 or less. This finding fell within the range of reported seroma rates of 2 to 50% from prior studies (2, 11, 18, 19). Our rates of breast fibrosis (*n* = 13, 32.5%) and indentation (*n* = 14, 35%) were similar to the 32% fibrosis rate reported in the Mussari et al. study (20). Despite being grade 3 or less, fibrosis and indentation both showed rising incidences as time progressed, likely reflecting the late manifestations of radiation injury. However, some early fibrosis may be attributed to the surgical procedure itself. Tuschy et al. reported that patients treated with IORT alone have a 50% decreased risk of developing higher-grade toxicities as compared to WBI (24). Unfortunately, our IORT and WBI group did not have an adequate sample size to justify a strong comparison. While our study supports the literature regarding the low toxicity profile of IORT, patient perception and satisfaction toward final aesthetic outcomes between IORT and WBI have yet to be delineated. Applying a more focused QoL assessment, such as the BreastQ survey, may better elucidate the cosmetic advantages of IORT.

Although the utilization of multiple QoL measures delivers a global understanding of the effects of IORT, this study has numerous limitations. The length of follow-up is sufficient to determine health-related QoL outcomes, but the oncologic effectiveness of IORT requires longer observation to boast comparable rates of local recurrence and disease-free survival as the TARGIT-A trial (5). Particularly regarding IORT-toxicity, more time is needed to assess long-term cardiopulmonary effects and development of secondary malignancies. In addition, the absence of a control group in this observational study limits our ability to compare the effects of IORT vs. WBI on QoL. Although we had nine patients who received IORT and WBI, the sample size was too small to serve as a robust control.

Given the novelty of IORT and the limited data surrounding this treatment modality, our study sought to elucidate its impact on patient QoL. Our results demonstrated that IORT could provide adequate local disease control while minimizing adverse effects based on subjective patient reports and objective clinician reports. Throughout the 2-year study, patients consistently reported strong emotional well-being and social support with only mild to moderate limitations on activities of daily living. At the same time, the severity and incidence of IORT-induced toxicities remained well within the range reported in prior literature. We advocate for the utility of IORT as an effective and patient-friendly alternative to WBI in the adjunctive treatment of early, localized breast cancer.

## Conclusion

IORT demonstrates favorable patient-reported QoL outcomes and low rates of toxicity with adequate local disease control at 2-year follow-up when implemented in early breast cancer.

## Author contributions

All authors listed have made a substantial, direct and intellectual contribution to the work, and approved it for publication.

### Conflict of interest statement

The authors declare that the research was conducted in the absence of any commercial or financial relationships that could be construed as a potential conflict of interest.

## Supplementary material

Supplemental Figure 1 IORT plus WBI Patient-Reported Health Status (EQ-5D). Patients reported scores between 0 and 1 across all symptoms through 1.5 years of follow up. Variations at the 2-year mark reflect the responses of a single patient.

Supplemental Figure 2 IORT plus WBI Patient-Reported Physical Well-Being (FACT-B). Patients reported scores between 0 and 2 across all symptoms through 1.5 years of follow up. Variations at the 2-year mark reflect the responses of a single patient.

Supplemental Figure 3 IORT plus WBI Patient-Reported Social Well-Being (FACT-B). Patients reported scores between 2 and 4 across all symptoms through 1.5 years of follow up, indicating “moderate” to “very much.” Variations at the 2-year mark reflect the responses of a single patient. Closeness to partner fluctuated throughout the follow up period.

Supplemental Figure 4 IORT plus WBI Patient-Reported Emotional/Functional Well-Being (FACT-B) and Fatigue Level (FACIT). Patients reported scores between 0 and 2 across the negative symptoms through 1.5 years of follow up, indicating “none” to “moderate,” and largely between 2 and 3 in the positive symptoms, indicating “moderate” to “quite a bit.” Variations at the 2-year mark reflect the responses of a single patient. Fatigue demonstrated a clear upward trend with time.

Supplemental Figure 5 IORT plus WBI Patient-Reported Symptoms (EORTC QLQ). Patients reported scores largely between 1 and 2 across all symptoms through 1.5 years of follow up. Variations at the two-year mark reflect the responses of a single patient. No consistent trends were observed.

## Abbreviations

IORT: Intraoperative Radiation Therapy
WBI: Whole Breast Irradiation
BCT: breast conservation therapy
QoL: Quality of Life
EQ-5D: Euro-QoL
FACT-B Version 4: Functional Assessment of Cancer Therapy-Breast
FACIT Fatigue Scale (Version 4): Functional Assessment of Chronic Illness Therapy
EORTC: European Organization for Research and Treatment of Cancer
QLQ-BR23: Quality of Life Questionnaire Breast Cancer Module
CTCAE: Common Terminology Criteria for Adverse Events
ADL: activities of daily living.
