# Supplementary figures and images for: A Prospective Analysis of Quality of Life and Toxicity Outcomes in Treating Early Breast Cancer With Breast Conservation Therapy and Intraoperative Radiation Therapy
Source: Front Oncol. 2018 Dec 3;8:545. doi: 10.3389/fonc.2018.00545 (PMC6287037; doi:10.3389/fonc.2018.00545)

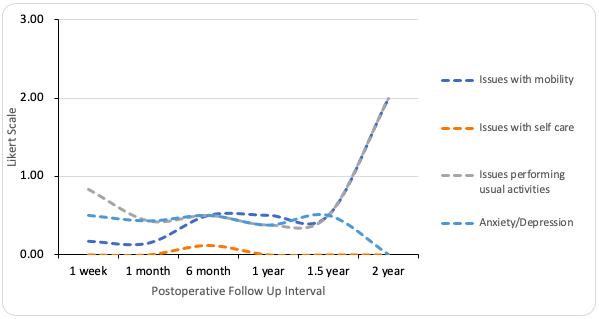

Supplement: Supplemental Figure 1 — IORT plus WBI Patient-Reported Health Status (EQ-5D). Patients reported scores between 0 and 1 across all symptoms through 1.5 years of follow up. Variations at the 2-year mark reflect the responses of a single patient. [file Image_1.jpeg]

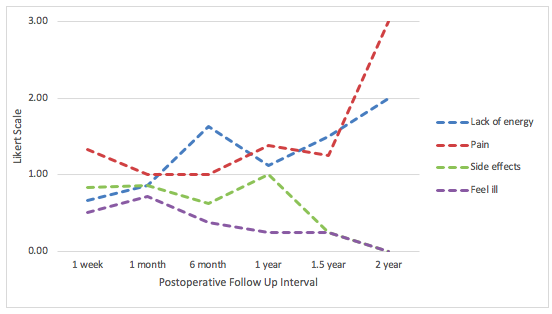

Supplement: Supplemental Figure 2 — IORT plus WBI Patient-Reported Physical Well-Being (FACT-B). Patients reported scores between 0 and 2 across all symptoms through 1.5 years of follow up. Variations at the 2-year mark reflect the responses of a single patient. [file Image_2.JPEG]

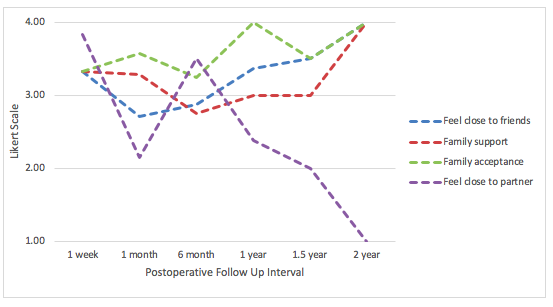

Supplement: Supplemental Figure 3 — IORT plus WBI Patient-Reported Social Well-Being (FACT-B). Patients reported scores between 2 and 4 across all symptoms through 1.5 years of follow up, indicating “moderate” to “very much.” Variations at the 2-year mark reflect the responses of a single patient. Closeness to partner fluctuated throughout the follow up period. [file Image_3.JPEG]

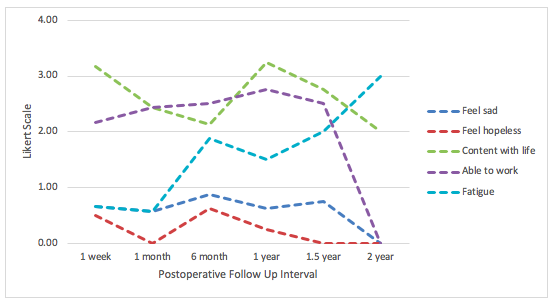

Supplement: Supplemental Figure 4 — IORT plus WBI Patient-Reported Emotional/Functional Well-Being (FACT-B) and Fatigue Level (FACIT). Patients reported scores between 0 and 2 across the negative symptoms through 1.5 years of follow up, indicating “none” to “moderate,” and largely between 2 and 3 in the positive symptoms, indicating “moderate” to “quite a bit.” Variations at the 2-year mark reflect the responses of a single patient. Fatigue demonstrated a clear upward trend with time. [file Image_4.JPEG]

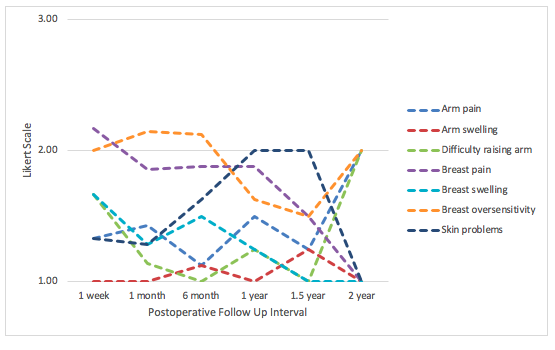

Supplement: Supplemental Figure 5 — IORT plus WBI Patient-Reported Symptoms (EORTC QLQ). Patients reported scores largely between 1 and 2 across all symptoms through 1.5 years of follow up. Variations at the two-year mark reflect the responses of a single patient. No consistent trends were observed. [file Image_5.JPEG]
